# Supplementary material for: Hot spot prediction in protein-protein interactions by an ensemble system
Source: BMC Syst Biol. 2018 Dec 31;12(Suppl 9):132. doi: 10.1186/s12918-018-0665-8 (PMC6311905; doi:10.1186/s12918-018-0665-8)
Supplement: Supplementary file 2 — All relevant MATLAB code. A simple Matlab implement of our predictor. (ZIP 19,742 kb) [file 12918_2018_665_MOESM2_ESM.zip › Additional file 2/Read ASA/ASA website/dbMPIKT/4NZWA.html]

 NetSurfP - Protein Surface Accessibility and Secondary Structure Predictions

  

|  |  |  |
| --- | --- | --- |
|  |  | NetSurfP - Protein Surface Accessibility and Secondary Structure Predictions  Technical University of Denmark |

  


---

```
# For publication of results, please cite:
# A generic method for assignment of reliability scores applied to solvent accessibility predictions.
# Bent Petersen, Thomas Nordahl Petersen, Pernille Andersen, Morten Nielsen and Claus Lundegaard
# BMC Structural Biology 2009, 9:51 doi:10.1186/1472-6807-9-51
#
# Column 1: Class assignment - B for buried or E for Exposed - Threshold: 25% exposure, but not based on RSA
# Column 2: Amino acid
# Column 3: Sequence name
# Column 4: Amino acid number
# Column 5: Relative Surface Accessibility - RSA
# Column 6: Absolute Surface Accessibility
# Column 7: Z-fit score for RSA prediction
# Column 8: Probability for Alpha-Helix
# Column 9: Probability for Beta-strand
# Column 10: Probability for Coil
E G  4NZWA                  1    0.927  72.971  -1.094   0.003   0.003   0.994
E A  4NZWA                  2    0.681  75.013  -2.031   0.058   0.017   0.925
E M  4NZWA                  3    0.534 106.793  -1.068   0.058   0.017   0.925
E A  4NZWA                  4    0.385  42.394  -2.177   0.018   0.019   0.964
E S  4NZWA                  5    0.550  64.448  -0.543   0.018   0.019   0.964
E H  4NZWA                  6    0.536  97.553  -0.924   0.018   0.019   0.964
E K  4NZWA                  7    0.421  86.538  -0.143   0.005   0.015   0.979
E S  4NZWA                  8    0.403  47.278   0.331   0.003   0.003   0.994
B P  4NZWA                  9    0.123  17.454  -0.868   0.600   0.003   0.397
E A  4NZWA                 10    0.342  37.666  -0.243   0.923   0.002   0.076
E D  4NZWA                 11    0.475  68.505   0.760   0.923   0.002   0.076
B I  4NZWA                 12    0.068  12.525   0.845   0.970   0.001   0.030
B V  4NZWA                 13    0.028   4.365   0.754   0.970   0.001   0.030
E K  4NZWA                 14    0.430  88.533   1.746   0.970   0.001   0.030
B N  4NZWA                 15    0.260  38.079   1.436   0.970   0.001   0.030
B L  4NZWA                 16    0.032   5.823   0.504   0.970   0.001   0.030
B K  4NZWA                 17    0.250  51.446   0.824   0.970   0.001   0.030
E E  4NZWA                 18    0.504  88.136   0.809   0.970   0.001   0.030
B S  4NZWA                 19    0.114  13.396  -0.144   0.970   0.001   0.030
B M  4NZWA                 20    0.065  13.087   0.473   0.970   0.001   0.030
E A  4NZWA                 21    0.382  42.140   0.632   0.923   0.002   0.076
E V  4NZWA                 22    0.335  51.566   0.746   0.858   0.002   0.139
B L  4NZWA                 23    0.079  14.520  -0.029   0.782   0.003   0.216
E E  4NZWA                 24    0.363  63.416  -0.068   0.622   0.015   0.363
E K  4NZWA                 25    0.644 132.471   0.248   0.321   0.003   0.675
E Q  4NZWA                 26    0.454  81.031  -0.751   0.109   0.005   0.886
E D  4NZWA                 27    0.645  93.017  -0.116   0.053   0.005   0.942
E I  4NZWA                 28    0.378  70.004  -1.083   0.053   0.005   0.942
E S  4NZWA                 29    0.557  65.327  -1.087   0.109   0.005   0.886
E D  4NZWA                 30    0.608  87.627  -1.102   0.406   0.004   0.590
E K  4NZWA                 31    0.665 136.852   1.026   0.782   0.003   0.216
E K  4NZWA                 32    0.377  77.590   0.526   0.858   0.002   0.139
B A  4NZWA                 33    0.220  24.255   0.355   0.923   0.002   0.076
E E  4NZWA                 34    0.482  84.170   0.764   0.923   0.002   0.076
E K  4NZWA                 35    0.450  92.627   1.535   0.970   0.001   0.030
B A  4NZWA                 36    0.080   8.827   0.377   0.970   0.001   0.030
B T  4NZWA                 37    0.124  17.199   0.693   0.970   0.001   0.030
E E  4NZWA                 38    0.450  78.650   0.773   0.988   0.000   0.012
E E  4NZWA                 39    0.329  57.441   0.721   0.988   0.000   0.012
B V  4NZWA                 40    0.029   4.380   0.806   0.988   0.000   0.012
B S  4NZWA                 41    0.124  14.580   0.177   0.970   0.001   0.030
E K  4NZWA                 42    0.507 104.310   1.238   0.970   0.001   0.030
B N  4NZWA                 43    0.146  21.404   0.341   0.970   0.001   0.030
B L  4NZWA                 44    0.042   7.672   0.483   0.970   0.001   0.030
E V  4NZWA                 45    0.377  57.930   1.031   0.970   0.001   0.030
E A  4NZWA                 46    0.371  40.884   0.870   0.938   0.007   0.055
B M  4NZWA                 47    0.033   6.603   0.459   0.938   0.007   0.055
B K  4NZWA                 48    0.216  44.493   1.069   0.938   0.007   0.055
E E  4NZWA                 49    0.440  76.851   0.483   0.911   0.033   0.057
B I  4NZWA                 50    0.119  21.960   1.114   0.831   0.044   0.125
B L  4NZWA                 51    0.043   7.837   0.070   0.701   0.107   0.192
E Y  4NZWA                 52    0.290  61.930   0.302   0.307   0.165   0.527
E G  4NZWA                 53    0.420  33.046  -0.359   0.053   0.043   0.903
E T  4NZWA                 54    0.526  73.012  -0.428   0.018   0.019   0.964
E N  4NZWA                 55    0.672  98.322  -0.823   0.016   0.005   0.979
E E  4NZWA                 56    0.563  98.409  -1.105   0.053   0.005   0.942
E K  4NZWA                 57    0.644 132.471   0.120   0.058   0.017   0.925
E E  4NZWA                 58    0.451  78.720   0.009   0.058   0.017   0.925
E P  4NZWA                 59    0.471  66.792  -0.077   0.058   0.017   0.925
E Q  4NZWA                 60    0.348  62.242   0.666   0.176   0.004   0.820
E T  4NZWA                 61    0.490  67.977   0.244   0.923   0.002   0.076
E E  4NZWA                 62    0.658 114.988   0.430   0.970   0.001   0.030
B A  4NZWA                 63    0.218  24.024   1.123   0.970   0.001   0.030
B V  4NZWA                 64    0.050   7.716   0.936   0.988   0.000   0.012
E A  4NZWA                 65    0.333  36.675   1.063   0.970   0.001   0.030
E Q  4NZWA                 66    0.399  71.244   1.392   0.988   0.000   0.012
B L  4NZWA                 67    0.049   8.899   1.027   0.970   0.001   0.030
B A  4NZWA                 68    0.057   6.259   0.633   0.988   0.000   0.012
E Q  4NZWA                 69    0.350  62.492   1.189   0.970   0.001   0.030
E E  4NZWA                 70    0.291  50.803   1.158   0.970   0.001   0.030
B L  4NZWA                 71    0.058  10.620   0.739   0.970   0.001   0.030
B Y  4NZWA                 72    0.115  24.490   1.094   0.858   0.002   0.139
E N  4NZWA                 73    0.460  67.373   0.473   0.782   0.003   0.216
E S  4NZWA                 74    0.305  35.781   0.194   0.600   0.003   0.397
E G  4NZWA                 75    0.354  27.836  -0.636   0.321   0.003   0.675
B L  4NZWA                 76    0.050   9.192   1.205   0.858   0.002   0.139
B L  4NZWA                 77    0.041   7.599   0.717   0.975   0.003   0.022
B S  4NZWA                 78    0.213  24.964   0.974   0.975   0.003   0.022
B T  4NZWA                 79    0.174  24.162   0.548   0.970   0.001   0.030
B L  4NZWA                 80    0.028   5.127   0.592   0.970   0.001   0.030
B V  4NZWA                 81    0.055   8.392  -0.550   0.970   0.001   0.030
E A  4NZWA                 82    0.321  35.385   0.921   0.923   0.002   0.076
B D  4NZWA                 83    0.182  26.284   0.495   0.858   0.002   0.139
B L  4NZWA                 84    0.050   9.192   0.831   0.502   0.002   0.495
E Q  4NZWA                 85    0.455  81.317   0.468   0.321   0.003   0.675
E L  4NZWA                 86    0.526  96.274   1.169   0.246   0.004   0.750
B I  4NZWA                 87    0.093  17.187   0.664   0.053   0.005   0.942
E D  4NZWA                 88    0.667  96.100   0.562   0.003   0.003   0.994
B F  4NZWA                 89    0.186  37.350   0.014   0.109   0.005   0.886
E E  4NZWA                 90    0.670 116.962   0.824   0.246   0.004   0.750
E G  4NZWA                 91    0.250  19.644   0.056   0.430   0.016   0.555
B K  4NZWA                 92    0.272  56.033   0.265   0.622   0.015   0.363
E K  4NZWA                 93    0.394  80.984   0.472   0.802   0.014   0.185
B D  4NZWA                 94    0.244  35.103   0.445   0.923   0.002   0.076
B V  4NZWA                 95    0.026   3.965   0.845   0.975   0.003   0.022
B A  4NZWA                 96    0.093  10.260   0.312   0.975   0.003   0.022
E Q  4NZWA                 97    0.278  49.704   1.561   0.992   0.000   0.007
B I  4NZWA                 98    0.031   5.754   0.622   0.992   0.000   0.007
B F  4NZWA                 99    0.048   9.634   0.773   0.988   0.000   0.012
B N  4NZWA                100    0.098  14.376  -0.090   0.970   0.001   0.030
B N  4NZWA                101    0.135  19.823   0.085   0.970   0.001   0.030
B I  4NZWA                102    0.036   6.605  -0.033   0.938   0.007   0.055
B L  4NZWA                103    0.070  12.835  -0.097   0.938   0.007   0.055
E R  4NZWA                104    0.348  79.784   0.736   0.717   0.014   0.269
E R  4NZWA                105    0.290  66.341   0.874   0.561   0.047   0.393
E Q  4NZWA                106    0.347  61.956   0.864   0.184   0.043   0.773
E I  4NZWA                107    0.322  59.551  -0.443   0.115   0.016   0.868
E G  4NZWA                108    0.716  56.373  -1.090   0.016   0.005   0.979
E T  4NZWA                109    0.632  87.658  -1.624   0.016   0.005   0.979
E R  4NZWA                110    0.563 128.835  -0.096   0.016   0.005   0.979
B T  4NZWA                111    0.282  39.044   0.048   0.016   0.005   0.979
B P  4NZWA                112    0.072  10.174  -0.137   0.113   0.043   0.844
B T  4NZWA                113    0.099  13.745   0.394   0.453   0.248   0.299
B V  4NZWA                114    0.074  11.358  -0.045   0.701   0.107   0.192
E E  4NZWA                115    0.451  78.772   1.238   0.911   0.033   0.057
B Y  4NZWA                116    0.144  30.794   1.210   0.879   0.010   0.111
B I  4NZWA                117    0.032   5.902   0.386   0.879   0.010   0.111
B C  4NZWA                118    0.186  26.171  -0.371   0.802   0.014   0.185
E T  4NZWA                119    0.522  72.360   0.457   0.622   0.015   0.363
B Q  4NZWA                120    0.205  36.667   1.404   0.181   0.016   0.803
B Q  4NZWA                121    0.196  35.023   0.929   0.782   0.003   0.216
E N  4NZWA                122    0.546  79.978   1.026   0.858   0.002   0.139
B I  4NZWA                123    0.044   8.066   0.709   0.970   0.001   0.030
B L  4NZWA                124    0.025   4.651   1.282   0.970   0.001   0.030
B F  4NZWA                125    0.205  41.103   0.100   0.988   0.000   0.012
B M  4NZWA                126    0.162  32.416   0.638   0.970   0.001   0.030
B L  4NZWA                127    0.031   5.658   0.436   0.970   0.001   0.030
B L  4NZWA                128    0.068  12.487  -0.694   0.923   0.002   0.076
E K  4NZWA                129    0.408  83.987   0.956   0.782   0.003   0.216
B G  4NZWA                130    0.182  14.331  -1.323   0.406   0.004   0.590
B Y  4NZWA                131    0.119  25.409   0.404   0.176   0.004   0.820
E E  4NZWA                132    0.612 106.847   0.070   0.053   0.005   0.942
E S  4NZWA                133    0.302  35.359   0.339   0.058   0.017   0.925
E P  4NZWA                134    0.383  54.419  -0.540   0.257   0.016   0.727
E E  4NZWA                135    0.423  73.968  -0.750   0.339   0.016   0.645
B I  4NZWA                136    0.069  12.783  -0.583   0.622   0.015   0.363
B A  4NZWA                137    0.059   6.491  -1.514   0.660   0.049   0.291
B L  4NZWA                138    0.151  27.648  -0.783   0.717   0.014   0.269
B N  4NZWA                139    0.178  26.118  -0.441   0.717   0.014   0.269
B C  4NZWA                140    0.041   5.784  -0.538   0.694   0.003   0.303
B G  4NZWA                141    0.077   6.036  -1.229   0.694   0.003   0.303
B I  4NZWA                142    0.143  26.474  -0.450   0.858   0.002   0.139
B M  4NZWA                143    0.035   6.963   0.018   0.938   0.007   0.055
B L  4NZWA                144    0.017   3.058   1.466   0.938   0.007   0.055
B R  4NZWA                145    0.149  34.144   0.472   0.938   0.007   0.055
B E  4NZWA                146    0.101  17.732  -0.370   0.879   0.010   0.111
B C  4NZWA                147    0.025   3.524   0.517   0.802   0.014   0.185
B I  4NZWA                148    0.038   7.085   0.141   0.622   0.015   0.363
E R  4NZWA                149    0.390  89.356   0.964   0.339   0.016   0.645
B H  4NZWA                150    0.184  33.506   0.647   0.430   0.016   0.555
E E  4NZWA                151    0.436  76.099   0.676   0.622   0.015   0.363
E P  4NZWA                152    0.410  58.236   0.566   0.717   0.014   0.269
B L  4NZWA                153    0.046   8.514  -0.057   0.751   0.050   0.199
B A  4NZWA                154    0.089   9.841   0.496   0.701   0.107   0.192
E K  4NZWA                155    0.434  89.315   1.682   0.649   0.163   0.188
B I  4NZWA                156    0.146  27.066   0.583   0.578   0.229   0.194
B I  4NZWA                157    0.086  15.892   0.671   0.352   0.332   0.316
B L  4NZWA                158    0.078  14.282  -0.434   0.148   0.418   0.435
E W  4NZWA                159    0.393  94.396   0.327   0.064   0.216   0.721
E S  4NZWA                160    0.407  47.736   0.025   0.184   0.043   0.773
E E  4NZWA                161    0.755 131.864   0.098   0.600   0.003   0.397
E Q  4NZWA                162    0.255  45.507   0.910   0.858   0.002   0.139
B F  4NZWA                163    0.036   7.165   0.071   0.923   0.002   0.076
E Y  4NZWA                164    0.296  63.277   0.844   0.938   0.007   0.055
E D  4NZWA                165    0.414  59.715   1.576   0.938   0.007   0.055
B F  4NZWA                166    0.052  10.356   0.949   0.938   0.007   0.055
B F  4NZWA                167    0.050  10.055   0.612   0.879   0.010   0.111
E R  4NZWA                168    0.462 105.821   1.611   0.879   0.010   0.111
B Y  4NZWA                169    0.188  40.154   0.248   0.879   0.010   0.111
B V  4NZWA                170    0.055   8.530   0.174   0.751   0.050   0.199
E E  4NZWA                171    0.514  89.848  -0.524   0.455   0.046   0.498
B M  4NZWA                172    0.253  50.645  -0.092   0.181   0.016   0.803
E S  4NZWA                173    0.469  55.014  -0.812   0.115   0.016   0.868
B T  4NZWA                174    0.167  23.205  -0.439   0.268   0.043   0.689
B F  4NZWA                175    0.072  14.470   0.213   0.605   0.105   0.290
E D  4NZWA                176    0.266  38.374   0.142   0.725   0.163   0.112
B I  4NZWA                177    0.076  14.115   0.913   0.725   0.163   0.112
B A  4NZWA                178    0.027   3.008   0.557   0.779   0.100   0.120
B S  4NZWA                179    0.106  12.423  -0.139   0.911   0.033   0.057
E D  4NZWA                180    0.272  39.195  -0.091   0.938   0.007   0.055
B A  4NZWA                181    0.036   3.934  -0.872   0.923   0.002   0.076
B F  4NZWA                182    0.087  17.441   0.088   0.970   0.001   0.030
E A  4NZWA                183    0.410  45.193   0.870   0.970   0.001   0.030
B T  4NZWA                184    0.127  17.615   0.426   0.970   0.001   0.030
B F  4NZWA                185    0.037   7.366   0.148   0.988   0.000   0.012
E K  4NZWA                186    0.329  67.675   1.389   0.988   0.000   0.012
E D  4NZWA                187    0.282  40.651   0.097   0.970   0.001   0.030
B L  4NZWA                188    0.049   9.009   0.341   0.970   0.001   0.030
B L  4NZWA                189    0.061  11.151  -0.125   0.923   0.002   0.076
E T  4NZWA                190    0.346  47.976  -0.275   0.782   0.003   0.216
E R  4NZWA                191    0.371  84.867   0.559   0.502   0.002   0.495
B H  4NZWA                192    0.147  26.685   0.257   0.109   0.005   0.886
E K  4NZWA                193    0.408  83.987   0.635   0.782   0.003   0.216
E L  4NZWA                194    0.643 117.678   0.395   0.879   0.010   0.111
B L  4NZWA                195    0.101  18.493   0.420   0.879   0.010   0.111
B S  4NZWA                196    0.033   3.868   0.003   0.938   0.007   0.055
E A  4NZWA                197    0.286  31.506   0.514   0.970   0.001   0.030
E E  4NZWA                198    0.537  93.866   1.284   0.970   0.001   0.030
B F  4NZWA                199    0.107  21.415   0.501   0.970   0.001   0.030
B L  4NZWA                200    0.052   9.576   0.279   0.970   0.001   0.030
E E  4NZWA                201    0.436  76.204   0.945   0.923   0.002   0.076
E Q  4NZWA                202    0.543  97.033   1.370   0.858   0.002   0.139
B H  4NZWA                203    0.200  36.289   0.428   0.694   0.003   0.303
B Y  4NZWA                204    0.076  16.284   1.367   0.923   0.002   0.076
E D  4NZWA                205    0.563  81.085   1.385   0.970   0.001   0.030
E R  4NZWA                206    0.374  85.623   1.970   0.970   0.001   0.030
B F  4NZWA                207    0.027   5.479   0.735   0.988   0.000   0.012
B F  4NZWA                208    0.062  12.504   1.062   0.988   0.000   0.012
E S  4NZWA                209    0.528  61.917   1.320   0.988   0.000   0.012
E E  4NZWA                210    0.273  47.746   1.900   0.988   0.000   0.012
B Y  4NZWA                211    0.039   8.420   0.523   0.988   0.000   0.012
B E  4NZWA                212    0.260  45.387   1.406   0.970   0.001   0.030
E K  4NZWA                213    0.439  90.405   1.644   0.970   0.001   0.030
B L  4NZWA                214    0.058  10.620   0.023   0.923   0.002   0.076
B L  4NZWA                215    0.070  12.762   0.141   0.782   0.003   0.216
E H  4NZWA                216    0.622 113.087   0.143   0.502   0.002   0.495
E S  4NZWA                217    0.390  45.696  -0.078   0.109   0.005   0.886
E E  4NZWA                218    0.665 116.193  -0.890   0.109   0.005   0.886
B N  4NZWA                219    0.264  38.591  -1.009   0.181   0.016   0.803
B Y  4NZWA                220    0.195  41.586   0.233   0.660   0.049   0.291
B V  4NZWA                221    0.126  19.305   0.099   0.831   0.044   0.125
B T  4NZWA                222    0.192  26.658   0.387   0.831   0.044   0.125
B K  4NZWA                223    0.142  29.168   0.590   0.879   0.010   0.111
B R  4NZWA                224    0.231  52.876   0.286   0.879   0.010   0.111
E Q  4NZWA                225    0.307  54.741   0.113   0.802   0.014   0.185
B S  4NZWA                226    0.068   7.958  -0.853   0.858   0.002   0.139
B L  4NZWA                227    0.050   9.082   0.706   0.923   0.002   0.076
E K  4NZWA                228    0.435  89.438   1.780   0.970   0.001   0.030
B L  4NZWA                229    0.099  18.182   0.401   0.970   0.001   0.030
B L  4NZWA                230    0.047   8.587   0.495   0.970   0.001   0.030
B G  4NZWA                231    0.169  13.285  -0.946   0.970   0.001   0.030
E E  4NZWA                232    0.399  69.723   1.097   0.970   0.001   0.030
B L  4NZWA                233    0.092  16.882   0.689   0.938   0.007   0.055
B L  4NZWA                234    0.094  17.230   0.015   0.782   0.003   0.216
B L  4NZWA                235    0.235  43.029  -0.127   0.502   0.002   0.495
E D  4NZWA                236    0.363  52.294   0.474   0.321   0.003   0.675
E R  4NZWA                237    0.455 104.103   0.364   0.600   0.003   0.397
E H  4NZWA                238    0.320  58.244  -0.607   0.782   0.003   0.216
B N  4NZWA                239    0.132  19.354  -0.419   0.782   0.003   0.216
B F  4NZWA                240    0.213  42.729   0.520   0.923   0.002   0.076
E T  4NZWA                241    0.494  68.490   1.147   0.970   0.001   0.030
B I  4NZWA                242    0.082  15.078   0.207   0.970   0.001   0.030
B M  4NZWA                243    0.090  18.009   0.040   0.970   0.001   0.030
E T  4NZWA                244    0.356  49.336   0.317   0.923   0.002   0.076
E K  4NZWA                245    0.387  79.668   1.331   0.879   0.010   0.111
B Y  4NZWA                246    0.112  23.849   0.383   0.802   0.014   0.185
B I  4NZWA                247    0.116  21.442  -0.694   0.522   0.016   0.462
E S  4NZWA                248    0.444  51.990  -0.833   0.181   0.016   0.803
E K  4NZWA                249    0.373  76.788   0.640   0.053   0.005   0.942
E P  4NZWA                250    0.487  69.134  -0.164   0.782   0.003   0.216
E E  4NZWA                251    0.564  98.443   0.566   0.782   0.003   0.216
B N  4NZWA                252    0.136  19.954   0.343   0.923   0.002   0.076
B L  4NZWA                253    0.029   5.328   0.584   0.970   0.001   0.030
E K  4NZWA                254    0.333  68.395   1.325   0.970   0.001   0.030
B L  4NZWA                255    0.092  16.790   0.842   0.970   0.001   0.030
B M  4NZWA                256    0.028   5.643   0.249   0.970   0.001   0.030
B M  4NZWA                257    0.047   9.505   0.858   0.970   0.001   0.030
E N  4NZWA                258    0.351  51.357   0.982   0.923   0.002   0.076
B L  4NZWA                259    0.092  16.827   0.258   0.858   0.002   0.139
B L  4NZWA                260    0.072  13.256  -0.393   0.694   0.003   0.303
E R  4NZWA                261    0.442 101.104   0.396   0.321   0.003   0.675
E D  4NZWA                262    0.391  56.285  -0.573   0.257   0.016   0.727
E K  4NZWA                263    0.618 127.225   0.054   0.181   0.016   0.803
E S  4NZWA                264    0.367  43.059  -0.851   0.181   0.016   0.803
E R  4NZWA                265    0.413  94.531   0.663   0.191   0.086   0.723
E N  4NZWA                266    0.373  54.593   0.297   0.148   0.418   0.435
B I  4NZWA                267    0.065  12.025   0.697   0.169   0.612   0.219
B Q  4NZWA                268    0.180  32.077   0.409   0.172   0.690   0.138
B F  4NZWA                269    0.107  21.535   0.120   0.169   0.612   0.219
B E  4NZWA                270    0.271  47.361   0.058   0.169   0.612   0.219
B A  4NZWA                271    0.055   6.028  -0.602   0.159   0.516   0.325
B F  4NZWA                272    0.096  19.187   0.254   0.079   0.592   0.329
B H  4NZWA                273    0.229  41.582   0.029   0.087   0.683   0.230
B V  4NZWA                274    0.054   8.269  -0.300   0.018   0.846   0.136
B F  4NZWA                275    0.075  15.053   0.106   0.018   0.846   0.136
B K  4NZWA                276    0.166  34.146   0.792   0.011   0.918   0.071
B V  4NZWA                277    0.029   4.473   0.840   0.011   0.918   0.071
B F  4NZWA                278    0.048   9.634   0.891   0.011   0.918   0.071
B V  4NZWA                279    0.043   6.640   0.027   0.018   0.846   0.136
B A  4NZWA                280    0.108  11.880  -0.803   0.022   0.552   0.426
B N  4NZWA                281    0.200  29.309   1.178   0.004   0.138   0.858
E P  4NZWA                282    0.581  82.430  -0.143   0.005   0.045   0.951
E N  4NZWA                283    0.669  97.956  -0.162   0.005   0.015   0.979
E K  4NZWA                284    0.417  85.818   0.182   0.016   0.005   0.979
B T  4NZWA                285    0.223  30.944  -0.214   0.109   0.005   0.886
E Q  4NZWA                286    0.651 116.269   1.026   0.858   0.002   0.139
E P  4NZWA                287    0.417  59.116   1.506   0.858   0.002   0.139
B I  4NZWA                288    0.022   3.996   0.907   0.923   0.002   0.076
B L  4NZWA                289    0.200  36.693   1.158   0.970   0.001   0.030
E D  4NZWA                290    0.562  81.013   2.008   0.970   0.001   0.030
B I  4NZWA                291    0.067  12.377   0.926   0.970   0.001   0.030
B L  4NZWA                292    0.041   7.580   0.294   0.975   0.003   0.022
E L  4NZWA                293    0.423  77.433   1.302   0.938   0.007   0.055
E K  4NZWA                294    0.552 113.546   1.557   0.858   0.002   0.139
B N  4NZWA                295    0.167  24.390   0.021   0.406   0.004   0.590
B Q  4NZWA                296    0.195  34.773   0.873   0.923   0.002   0.076
E A  4NZWA                297    0.653  71.983   0.981   0.970   0.001   0.030
E K  4NZWA                298    0.299  61.587   2.092   0.970   0.001   0.030
B L  4NZWA                299    0.019   3.497   1.354   0.970   0.001   0.030
B I  4NZWA                300    0.145  26.751   1.134   0.988   0.000   0.012
E E  4NZWA                301    0.554  96.749   1.936   0.970   0.001   0.030
B F  4NZWA                302    0.082  16.397   0.922   0.970   0.001   0.030
B L  4NZWA                303    0.052   9.430   0.531   0.923   0.002   0.076
E S  4NZWA                304    0.603  70.625   1.174   0.858   0.002   0.139
E K  4NZWA                305    0.678 139.485   1.450   0.694   0.003   0.303
B F  4NZWA                306    0.054  10.838   1.031   0.321   0.003   0.675
E Q  4NZWA                307    0.551  98.426   0.455   0.109   0.005   0.886
E N  4NZWA                308    0.404  59.219  -0.348   0.053   0.005   0.942
E D  4NZWA                309    0.727 104.717  -1.112   0.016   0.005   0.979
E R  4NZWA                310    0.434  99.317  -0.169   0.053   0.005   0.942
E T  4NZWA                311    0.482  66.853  -1.049   0.058   0.017   0.925
E E  4NZWA                312    0.592 103.492  -0.558   0.115   0.016   0.868
E D  4NZWA                313    0.331  47.639  -0.600   0.181   0.016   0.803
E E  4NZWA                314    0.679 118.621   0.350   0.339   0.016   0.645
E Q  4NZWA                315    0.473  84.496   1.197   0.430   0.016   0.555
B F  4NZWA                316    0.076  15.233   0.284   0.622   0.015   0.363
E N  4NZWA                317    0.495  72.527   0.859   0.879   0.010   0.111
E D  4NZWA                318    0.592  85.250   0.704   0.923   0.002   0.076
B E  4NZWA                319    0.258  45.125   0.318   0.970   0.001   0.030
B K  4NZWA                320    0.173  35.504   0.687   0.970   0.001   0.030
E T  4NZWA                321    0.453  62.831   0.875   0.970   0.001   0.030
E Y  4NZWA                322    0.335  71.632   1.769   0.970   0.001   0.030
B L  4NZWA                323    0.047   8.569   0.797   0.970   0.001   0.030
B V  4NZWA                324    0.079  12.065   0.202   0.988   0.000   0.012
E K  4NZWA                325    0.524 107.869   1.709   0.970   0.001   0.030
E Q  4NZWA                326    0.271  48.329   1.438   0.970   0.001   0.030
B I  4NZWA                327    0.078  14.485   0.020   0.923   0.002   0.076
E R  4NZWA                328    0.537 123.019   1.248   0.858   0.002   0.139
E D  4NZWA                329    0.712 102.614   1.517   0.600   0.003   0.397
E L  4NZWA                330    0.242  44.310   0.022   0.246   0.004   0.750
E K  4NZWA                331    0.880 181.057   1.079   0.003   0.003   0.994

Explain the output.  Go back.
```

---
